# Pattern of healthcare resource utilization and direct costs associated with manic episodes in Spain

**DOI:** 10.1186/1471-244X-10-31

**Published:** 2010-04-28

**Authors:** Monica Tafalla, Luis Salvador-Carulla, Jerónimo Saiz-Ruiz, Teresa Diez, Luis Cordero

**Affiliations:** 1 Medical Department, AstraZeneca, Madrid, Spain; 2 PSICOST Scientific Research Association, Cádiz, Spain; 3 Ramon y Cajal Hospital and University of Alcalá, Madrid, Spain; 4 Columbia University, Department of Psychiatry, New York, USA

## Abstract

**Background:**

Although some studies indicate that bipolar disorder causes high health care resources consumption, no study is available addressing a cost estimation of bipolar disorder in Spain. The aim of this observational study was to evaluate healthcare resource utilization and the associated direct cost in patients with manic episodes in the Spanish setting.

**Methods:**

Retrospective descriptive study was carried out in a consecutive sample of patients with a DSM-IV diagnosis of bipolar type I disorder with or without psychotic symptoms, aged 18 years or older, and who were having an active manic episode at the time of inclusion. Information regarding the current manic episode was collected retrospectively from the medical record and patient interview.

**Results:**

Seven hundred and eighty-four evaluable patients, recruited by 182 psychiatrists, were included in the study. The direct cost associated with healthcare resource utilization during the manic episode was high, with a mean cost of nearly €4,500 per patient, of which approximately 55% corresponded to the cost of hospitalization, 30% to the cost of psychopharmacological treatment and 10% to the cost of specialized care.

**Conclusions:**

Our results show the high cost of management of the patient with a manic episode, which is mainly due to hospitalizations. In this regard, any intervention on the management of the manic patient that could reduce the need for hospitalization would have a significant impact on the costs of the disease.

## Background

Bipolar disorder is a mood disorder characterized by extreme mood swings that cause recurrent episodes of mania or hypomania and depression [1]. Historically, it was called "circular madness" and "manic-depressive psychosis". According to DSM-IV-TR, two major categories of bipolar disorder exist: bipolar I disorder, in which patients have had at least one episode of mania, some have had previous depressive episodes, and most will have subsequent manic, depressive, hypomanic or mixed episodes; and bipolar II disorder, in which patients exhibit or have a history of major depressive episodes and hypomanic, but not manic, episodes [2].

In Europe, the estimated annual prevalence of bipolar disorder ranges from 0.2 to 1.1% with a median of 0.9%, i.e., 2.4 million people are affected by the disorder [3]. In Spain, using data on lithium consumption, the prevalence of bipolar I disorder has been estimated at 70 cases per 100,000 inhabitants [4], a figure that, because of the method used, underestimates the true prevalence of the disorder. Bipolar disorder is not only common, but is also an important cause of disability; it exhibits frequent psychiatric comorbidity, is associated with a high frequency of suicide, has a large impact on the functioning and well-being of the individual, and places a considerable economic burden on the individual and society [5-13].

According to the World Health Organization, bipolar disorder is the sixth leading cause of disability worldwide among persons aged 15 to 44 years [5], and the third among mental illnesses (after major depression and schizophrenia). The data provided by this organization in 2005 attributed more than thirty percent of all years lived with disability to neuropsychiatric disorders [6]. In addition, patients with bipolar disorder have high psychiatric and medical comorbidity; in studies conducted in Europe, nearly all patients with bipolar I disorder had a history of having suffered another axis I disorder in their lifetime, more than two thirds had a history of one or more anxiety disorders and 70% had a history of a substance abuse disorder [7]. The lifelong risk of suicide in bipolar disorder is up to 20 times higher than in the general population [8-10]. Several studies have shown that even in less symptomatic patients (i.e. sub-threshold symptoms present), bipolar disorder causes a significant impairment of the functioning and well-being of the individual [11-13].

The studies conducted to date have identified high resource utilization and costs in bipolar disorders that were the highest among psychiatric disorders [14,15]. A prevalence cost study conducted in the USA estimated that the total cost of bipolar disorder in 1991 was $45 billion [16]. Another study on incident cases in 1998 estimated the lifetime cost of bipolar disorder at $24 billion [17]. Another study has recently been published in which the treatment costs of bipolar disorder in the USA in 2002 were estimated. The results were $12,797 and $6,581 for the mean charge and reimbursement per patient-year, respectively. In this study, 33% of the treatment cost was attributed to bipolar disorder and the remaining 67% to associated comorbidity [18]. In Australia, the excess cost of bipolar disorder in 2004 was estimated at US$4-5 billion [19].

In Europe, only four studies have assessed the cost of bipolar disorder [15]: two in France focusing on manic episodes [20,21], one in the Netherlands [22] and another in the United Kingdom [23], these last two focusing on bipolar disorder. The results differ greatly between the European and US studies; in the UK study, direct costs were estimated at approximately €285 million, compared to the equivalent of €3 billion in the USA [16]. The differences between the studies in Europe are also large with, for instance, direct costs that range from €700 to €24,000 per patient depending on the study [20-23]. These differences reflect differences in the management of the disease (mostly rates and duration of hospitalization) as well as the different perspectives in research question and methodologies.

No study is available on the costs of bipolar disorder in Spain. Very recently published data from a subsample of a pan-European study indicate that bipolar disorder causes high healthcare resource utilization in the Spanish setting, although no cost estimate was provided [24]. In another estimate of the cost of disorders of the brain in Europe [25], it was shown that bipolar disorder is the mental disorder generating the highest costs in Spain (5,807 €PPP 2004 (Purchasing power parity) per patient versus 5,082 for schizophrenia and 3,445 for depression). The aim of the present study was to evaluate healthcare resource utilization and the associated direct cost in patients with manic episodes in our setting.

## Methods

An observational study with retrospective data collection was carried out in a sample formed by consecutive patients with a DSM-IV diagnosis of bipolar type I disorder with or without psychotic symptoms visiting psychiatrist outpatient offices in Spain. The selected patients were aged 18 years or older, were having an active manic episode at the time of inclusion and were in contact with specialized care (public or private) for this reason in Spain during the reference period of April 2005 to March 2006.

Patients could be included at any time during the course of a manic episode, and information regarding the period between the onset of symptoms of that episode to the time of inclusion was collected retrospectively at the recruitment moment. A second phase of data collection was performed when the episode had ended. A maximun period of four months was defined in the protocol as sufficient for complete remission of the episode, and patients with no remission at four months were no longer followed. The study was carried out under real-world clinical practice conditions in an outpatient setting and information was collected in a case report form designed for this purpose.

The study was evaluated and approved by the ethics committee of Hospital Clínico San Carlos de Madrid and carried out in accordance with the ethical recommendations for clinical research contained in the Declaration of Helsinki and Good Clinical Practice guidelines. Written informed consent was obtained from all patients prior to their inclusion in the study.

Information was collected on each patient characteristics (sociodemographics, personal and family medical history), disease characteristics (duration of compatible symptoms, diagnosis, previous episodes) and current episode characteristics. All healthcare resources consumed during the current episode (drugs, outpatient and hospital care) were recorded using the medical history and patient interview as sources of data. The evaluator made a judgment about the relationship of each resource consumed with the patient's disease. Information was also collected on the existence of any legal or judicial problems during the manic episode, although their costs were not estimated.

The unit costs assigned to the healthcare resource utilization recorded for each patient were obtained from a healthcare unit costs database [26]. These unit costs were then updated to the year 2007 according to the corresponding inflation rate, 12.5% [27]. In addition, the costs of psychologist visits and group psychotherapy were obtained by calculating the average value of the fee lists published by several official psychologists' associations on the minimum cost of a patient visit. Finally, the cost of prescription drugs was obtained from the retail price of each individual drug including VAT published by the General Board of the Spanish Association of Official Pharmacists [28]. The cost per mg was then calculated to assign the actual cost of the drug to the dose prescribed and treatment duration in each patient. A list of all costs, expressed in 2007 Euros, is shown in Table 1.

**Table 1:** Unit cost per healthcare resource used and source of estimate

Resource	Source of estimate	Cost (€, 2007)
Hospitalization		

Psychiatric hospital stay/day	Soikos (2004) & INE (2007)	240.27

Primary care		

Primary care physician visit	Soikos (2004) & INE (2007)	15.67
Community-based visiting nurse service	Soikos (2004) & INE (2007)	15.65
Lithium determination	Soikos (2004) & INE (2007)	9.02
Group psychotherapy	Official Psychologist Associations of Cataluña, Castilla la Mancha, Cantabria, Las Palmas, Barcelona	24.86
Outpatient emergency dept. visit	Soikos (2004) & INE (2007)	110.94

Specialized care		

Psychiatrist visit	Soikos (2004) & INE (2007)	39.99
Nonpyschiatric specialist visit	Soikos (2004) & INE (2007)	79.46
Psychologist visit	Official Psychologist Associations of Cataluña, Castilla la Mancha, Cantabria, Las Palmas, Barcelona	50
Hospital emergency dept. visit	Soikos (2004) & INE (2007)	120.21

Statistical analysis was performed by describing demographic variables, patient disease and resource utilization. Quantitative variables (e.g., age, disease duration, absolute frequency of resource utilization) were described by their mean values and standard deviations. Categorical variables (e.g., gender, comorbidity, presence of a specific number of hospitalizations or other resource utilization) were described by their absolute and relative frequencies.

In addition, to evaluate how sociodemographic or clinical characteristics affected resource utilization, exploratory bivariate analyses were used to compare resource utilization according to the values that could be taken by the different variables. To evaluate the significance of the difference, Student's t test or the Wilcoxon signed rank test was used for quantitative variables and the chi-squared test for Fisher's exact test for categorical variables. All statistical tests were two-tailed and were considered significant if p < 0.05. Due to the exploratory nature of these analyses, no correction for multiple comparisons was used.

## Results

Nine hundred and ten patients, evaluated by 182 psychiatrists, were included in the study. Of these, 126 patients' data were considered non-evaluable because of missing or inconsistent values and then excluded from the data base.

Demographic characteristics of evaluable patients are shown in Table 2. Most patients lived with their partner and were employed, although a substantial percentage (21.3%) were on disability leave. Most of the sample lived in small urban areas with populations between 10,000 and 100,000 or medium-sized urban areas with populations between 100,000 and 1,000,000.

**Table 2:** Demographic characteristics

Characteristic	N	
Gender, n (%)	761	
Male		343 (45.2)
Female		418 (54.8)

Age, years, mean ± SD		
Total	761	43.5 ± 12.1
Male	343	41.9 ± 12.2
Female	418	44.8 ± 11.8

Educational status, n (%)	784	
No studies		73 (9.3)
Primary school		340 (43.4)
Secondary school		256 (32.6)
University		115 (14.7)

Marital status, n (%)	762	
Married or cohabiting		337 (44.2)
Previously married		136 (17.9)
Never married		289 (37.9)

Employment status, n (%)	762	
Paid employment		202 (26.5)
Unemployed		81 (10.6)
Retired		81 (10.6)
Housewife		130 (17.1)
Student		24 (3.1)
Sick leave		66 (8.7)
Work disability		162 (21.3)
Other		16 (2.1)

Area of residence, n (%)	760	
Rural		156 (20.5)
Small urban		259 (34.1)
Medium urban		255 (33.6)
Large urban		90 (11.8)

Living situation, n (%)	779	
Lives alone		98 (12.6)
Lives with someone		681 (87.4)

SD: standard deviation; N: number of evaluable cases

Clinical characteristics of the patients are described in Table 3. The first professional consulted by patients for the initial episode was the psychiatrist in the majority of cases, and this episode required hospital admission in 23.6% of cases. Only 4.1% of patients were newly diagnosed. In the twelve months prior to the current episode, only 32.8% of patients had been free from symptoms. In this period, 30.9% of patients had had one episode of mood disorder and 20.2% two episodes. Of the total sample, 6.5% met the criteria for rapid cycling (four or more episodes a year). Up to 28.1% had a previous suicide attempt. Less than 10% of patients had never been hospitalized from the onset of their disease to the time of inclusion in the study, and 25.6% had been admitted more than 5 times during this period. From the physician's perspective, up to 31% of patients had shown low adherence to the previous visit schedule, and up to 38.4% had shown low adherence to previously prescribed treatments.

**Table 3:** Clinical characteristics

Characteristic	N	
First episode		
Age at onset of BD, years, mean ± SD	737	29.0 ± 10.3
First manic/mixed episode, n (%)	748	479 (64.0)
Time since first episode at diagnosis, years, mean ± SD	734	2.4 ± 5.6
Type of first contact with healthcare sector, n (%)		
Psychiatrist		300 (41.5)
Hospitalization		171 (23.6)
Primary care		140 (19.3)
Emergency department	724	72 (9.9)
Other		41 (5.7)

Current episode		
Total duration, days, mean ± SD	708	76.4 ± 43.0
Clinical status prior to current episode, n (%)	741	
Euthymia		567 (76.5)
Depression		174 (23.5)

Suicide	744	
Presence of previous attempts, n (%)		209 (28.1)

Psychiatric comorbidity^1^, n (%)	749	
Any concomitant psychiatric disorder		410 (54.7)
Substance abuse/dependence disorder		200 (26.7)
Anxiety disorders		119 (15.9)
Personality disorder		107 (14.3)
Impulse control disorder		65 (8.7)
Eating behavior disorder		36 (4.8)
Other disorders		28 (3.7)

High adherence to previous visits schedule, n (%)	743	513 (69.0)

High adherence to previous treatment, n (%)	744	458 (61.6)

^1^Patients could have more than one disorder

SD: standard deviation; N: number of evaluable cases; BD: bipolar disorder

Mean total duration of the current manic episode was 76.4 days (SD: 43). Prior to the current episode, 76.5% of patients were in an euthymic state and up to 23.5% were in a depressed mood state.

The information on resource utilization is shown in Tables 4. Half of the sample studied required hospitalization, which was in a general hospital in 71.8% of the cases. The mean length of hospital stay was 22.9 days (SD: 15.5), and bipolar disorder was the primary reason for admission in 93% of the cases. The mean number of visits to the primary care physician during the episode was 1.9 and 1.6 to the community-based nurse service. The specialist was visited a mean of 5.7 times during the episode. Patients with four or more episodes in the previous year had more lithium determinations (1.1 vs. 2.7; p = 0.0003), and made more visits to outpatient emergency services (0.4 vs. 1.4; p < 0.0001) due to their current manic episode. Patients who had never been married (p = 0.424), were from a rural setting (p = 0.0048) and had longer disease duration (p for trend = 0.0137) were hospitalized more frequently. Patients who lived alone made more visits to the psychiatrist (8.6 vs. 5.3 times, p = 0.0032). The presence of a history of suicide attempt was associated with a higher number of visits to the psychologist (1.1 vs. 0.6, p = 0.02), non-psychiatrist specialist (0.6 vs. 0.1, p < 0.0001) and hospital emergency department (1.6 vs. 0.8, p = 0.0005). Finally, the absence of psychiatric comorbidity was associated with a higher number of visits to the psychologist. The pharmacological treatment received by patients over the course of their episode consisted of antipsychotics, mood stabilizers and anxiolytics with frequencies of 94.6%, 83.9% and 55.2%, respectively.

**Table 4:** Resource utilization in a cohort of patients with bipolar disorder who had a manic episode: hospitalizations

Characteristics	N	
Required hospitalization, n (%)	782	391 (50)

Days of hospitalization^1^, mean ± SD	383	22.9 ± 15.5

Cause of hospitalization^1^, n (%)	389	
Current manic episode^2^		362 (93.0)
Psychiatric comorbidity		14 (3.6)
Other causes		13 (3.4)

Type of hospital^1^, n (%)	386	
Monographic		109 (28.2)
General		277 (71.8)

^1^Calculated over the number of evaluable patients who required hospitalization

^2^Includes 4 cases in which the reason for hospitalization was attributed to both the manic episode and the comorbidity

SD: standard deviation; N: number of evaluable cases

**Figure 1:**
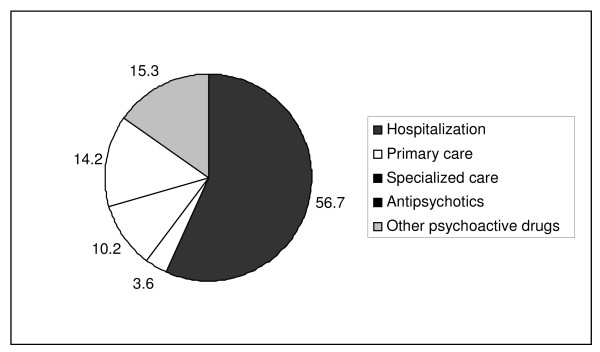
**Percent distribution of direct costs associated with the management of a patient with a manic episode (N = 708)**.

The mean total cost of the manic episode in the sample studied was €4,345. Of this cost, 56% corresponds to hospitalization, 10% to specialist care (mainly from psychiatrist visits, with a mean of 6), 14% to antipsychotics and 15% to other psychoactive drugs (Figure 1). The direct costs associated with the resources used are shown in Table 5.

**Table 5:** Direct costs associated with the management of a patient with a manic episode (N = 708)

Resource	Number of times during manic episode	Cost (€, 2007)	
	Mean ± SD	Mean ± SD	95% CI

Hospitalization			

Psychiatric hospital stay/day		2462 ± 3424	2210 - 2715

Primary care			

Primary care physician visit	1.9 ± 6.0	31 ± 97	24 - 39
Nurse visit	1.6 ± 4.3	21 ± 56	17 - 25
Lithium determination	1.3 ± 3.1	12 ± 29	10 - 14
Group psychotherapy	0.6 ± 10.8	25 ± 454	0 - 58
Outpatient emergency dept. visit	0.6 ± 1.8	68 ± 213	52 - 84

Specialized care			

Psychiatrist visit	5.7 ± 10.3	258 ± 429	227-290
Nonpsychiatric specialist visit	0.3 ± 1.4	22 ± 113	14 - 30
Psychologist visit	0.8 ± 2.4	39 ± 124	30 - 48
Hospital emergency dept. visit	1.0 ± 2.8	124 ± 352	98 - 150

Pharmacological treatment			

Antipsychotics	751 (94.6)	617 ± 656	569 - 665
Other psychoactive drugs^1^	Range from 52 (anticholinergics) to 666 (mood stabilizers	666 ± 679	616 - 716

**Total cost**		4345 ± 4019	4048 - 4641

SD: standard deviation; CI: confidence interval; ^1^Other psychoactive drugs: Includes the cost associated with mood stabilizers, anxiolytics/hypnotics, antidepressants and anticholinergics.

## Discussion

This naturalistic study shows that management of a manic episode in the Spanish setting is associated with high healthcare resource utilization, particularly in terms of hospitalization and specialized care in the form of frequent psychiatrist visits. The direct cost associated with healthcare resource utilization is high, with a mean cost of nearly €4,500 per patient, of which approximately 55% corresponds to the cost of hospitalization, 30% to the cost of psychopharmacological treatment and 10% to the cost of specialized care.

To our knowledge, this is the first study of these characteristics conducted in Spain, so it is not possible for us to put our results in perspective within our setting. The results obtained in the Spanish subsample of 312 patients within the pan-European EMBLEM study, a long-term observational study of acute patients undergoing treatment for mania, have recently been published [24]. Although this subanalysis of the EMBLEM study is very limited with regard to healthcare resource utilization, it does appear to indicate a significant utilization of some healthcare services by these patients in the year prior to inclusion in the study. However, the methodology used, which was limited to the use of a small number of healthcare resources (i.e., hospitalizations and outpatient psychiatric visits) in the year prior to the episode, prevents comparison with our results since they would not correspond to the resource utilization associated with a manic episode.

Very similar to our study in terms of objectives was a study conducted in France that evaluated the direct cost of treatment of manic episodes during a three-month period following hospitalization [20]. The cost, in 1999 values, was much higher than in our study, €22,297 per episode, and 98% corresponded to hospitalization [20]. At least in Europe, the cost of hospitalization is the most significant portion of the direct costs of bipolar disorder [29], and furthermore, the largest part of these costs of hospitalization is attributable to bipolar I disorder [30]. Therefore, as indicated by the results of Olié & Lévy's [20] and our study, hospitalization is key in the cost of management of patients with bipolar I disorder and, more specifically, of the manic episode. Irrespective of possible differences in the unit cost per resource, there are several important differences related to hospitalization in the French study that could explain the differences in the cost of the manic episode between the two studies. Only hospitalized patients were included in the French study, whereas in our study, more than 50% of patients were not hospitalized. Furthermore, the mean duration of hospitalization was 36 days in the French study versus 23 days in our study, and follow-up was for 90 days in the French study versus a mean duration of the episode of 76 days in our study. Although these differences could be attributed to variability in medical practices and resource availability in the two countries, it should be noted that differential diagnosis between mania and hypomania in DSM-IV-TR includes use of hospital resources as a diagnostic criterion, which constitutes a peculiarity within the field of medical nosology. In any case, the contribution of hospitalization to the cost of manic episodes is very significant, independent of the geographical area. Thus, costs of hospitalization also account for the largest proportion of the total costs of bipolar disorder in Australia (70% of the excess healthcare costs of bipolar disorder are due to hospital admissions) [19] and in the United States, where 36% of the annual cost of patients with bipolar I disorder is due to hospitalization for privately insured patients [31].

After hospitalization, the next greatest cost in our study is the cost of psychopharmacological treatment (30% of total cost). The cost of antipsychotic treatment represents 50% of this pooled cost. The pattern of psychopharmacological treatment in our study, with use of antipsychotics and mood stabilizers in 95% and 84% of patients, is practically superimposable on that described in the previously mentioned study of Olié & Lévy [20] conducted in France. However, the cost of medication in the latter study was a minimal proportion (0.3%) of the cost of treatment in the three months following the manic episode. This was probably due to the disproportionate (for the previously explained reasons) importance of hospitalization in this study and the predominant use of conventional antipsychotics. However, in the study of privately insured patients in the United States [31], the cost of psychopharmacological treatment was 13% of the total cost.

Our study has a number of important limitations. First, convenience sampling was used, so this sample is not representative of patients with a manic episode in Spain. While it is true that the overall demographic and clinical characteristics of the patients in our study are very similar to those of the Spanish sample in the EMBLEM study [24], patients from the rural setting may be underrepresented in both studies.

The problem of lack of representativeness affects most cost studies carried out using a "bottom-up" methodology (activity-based costing method that assess the amount of each resource that is used to produce an individual healthcare service and then assigns costs accordingly to generate aggregate costs for a healthcare system). The main advantage is being able to trace the contribution of each element of an organization to the cost of an individual healthcare service, which allows for better cost management when is particularly relevant for assessing the cost of individual services within complex integrated healthcare systems, as the Spanish one. Additionally, the type of information obtained through a "bottom-up" is very relevant for its inclusion in cost-effectiveness modeling studies using combined or cross-national synthesis designs [32]. On the other side, a "top-down" approach (using relative value units, hospitals days, or some other metric to assign total costs for a healthcare system to individual services) could be useful as well in order to assess local cost variation. From our point of view, an utilization of both methods could be advantageous because different methods can serve different purposes, and finally are complementary [33].

The study protocol did not define a standardized method for patient diagnosis, but followed psychiatrist opinion, and this could affect the validity of diagnosis, although we presume that the case of mania could be not as affected as other mental diagnoses. Moreover, for public health decisions the relevant cost of a disease comes from the population considered by the specialists as suffering from the disease.

Also, due to the descriptive retrospective study design, no information can be provided on some predictors of higher cost, such as treatment adherence or persistence on treatment. It has been shown that a better adherence associates with a lower cost in the long term treatment [34].

With regard to the method used for cost allocation, it is important to point out two limitations in our study. First, the healthcare costs database used, SOIKOS, has been the standard in Spain for several years. This private database is based on the information gathered from government agency publications, published studies and literature reviews, among others. Its very nature means that the costs provided have not been sufficiently verified or have rapidly become outdated. Second, adjustment of these costs according to inflation is a method that has been questioned on some occasions, a factor that should also be taken into account. Ideally, to overcome these limitations, a single nationwide database, mainly related to public costs as Spain has a public health care system funded by public taxes, would be needed to perform a cost allocation closer to the reality of our healthcare system.

On the other hand, it should be stressed that a more conservative perspective was adopted in this study, and only direct costs were analyzed. No costs were allocated to disease associated mortality, lost productivity, use of the legal or penal system or the associated family burden, in spite of the relative importance of these costs. Regarding the impact of legal problems, reports about the importance of mental health problems in the prison and jail inmates in the USA, estimate that up to 50% of inmates with mental problems report symptoms of mania [35].

Of the estimated $45 billion total cost of bipolar disorder in the United States in 1991 [16], more than 80% was due to indirect costs, a very similar proportion to that described in another study in the Netherlands [22]. Similarly, of the total excess cost of bipolar disorder in Australia, the largest proportion (85%) was due to individual expenses; 60% of these were due to absenteeism from work and 39% to "presenteeism" (present at work but not functioning efficiently). This large impact on productivity extends beyond the manic episode. In a prospective study six months after discharge that evaluated patients who had been hospitalized after a manic episode, even though 80% were practically symptom free, only 43% were employed and only 21% were working at their expected level of employment [12].

The work disability rate found in our sample is similar to that reported in a study on the employment status of persons with severe chronic mental illnesses based on the national survey on disability conducted in 1999 (20.36%) [36]. However, the employment rate of the persons with mania included in our study was lower than the employment rate of persons with mental disorders reported in the ESEMeD study in Spain (36.7%) [37]. Furthermore, access to sheltered employment conditions is considerably lower in patients with bipolar disorder than in other severe mental disorders. In Catalonia, only 7% of persons in sheltered employment had bipolar disorder, compared to the 62% with schizophrenia or 8% with borderline personality disorder (MHEEN-II, 2007). These data indicate that the employment status of persons with bipolar type I disorder requires a specific approach in Spain.

Although it has been pointed that other health economic appraisals can help more policy makers determine the maximum societal benefit that can be achieved, given a finite amount of resources [38], the cost of illness studies are still useful for both clinicians and health authorities to better understand the main sources of cost and identify those aspects that can be subject of interventions and whose efficiency can be analyzed.

## Conclusions

Our study is the first to study resource utilization and costs associated with manic episodes in Spain using a bottom-up approach. Like other studies conducted in Europe and elsewhere, it shows the high cost of management of the patient with a manic episode, which is mainly due to hospitalizations. In this regard, any intervention in the management of the manic patient that reduces the need for hospitalization (e.g., improved preventive pharmacological measures or measures that improve the family or social support of the patient with bipolar disorder) would have a significant impact on the costs of the disease.

## Competing interests

This study was funded by AstraZeneca Farmacéutica Spain in 2005. MT, TD and LC are full-time employees of AstraZeneca. JS has been a consultant to Astra-Zeneca, BristolMyers-Squibb, Lilly, GlaxoSmithKline, Lundbeck, Pfizer, Servier, Janssen, and Wyeth; and has received research grants from Lilly, Astra-Zeneca, Janssen, BristolMyers-Squibb and Wyeth. LS had previously been a consultant to Astra-Zeneca, BristolMyers-Squibb, Lilly and Janssen. But during the last three years he has not signed any contract or received research grants from pharmaceutical companies.

## Authors' contributions

All authors participated in the design of the study, the statistical analysis plan and the interpretation of the data. MT conceived of ths study and participated in its coordination. All authors read and approve the final manuscript.

## Pre-publication history

The pre-publication history for this paper can be accessed here:

http://www.biomedcentral.com/1471-244X/10/31/prepub
